# A Conductive, Photothermal and Antioxidant ε-Poly-L-Lysine/Carbon Nanotube Hydrogel as a Candidate Dressing for Chronic Diabetic Wounds

**DOI:** 10.3390/polym18030332

**Published:** 2026-01-26

**Authors:** Jinqiang Zhu, Wenjun Qin, Bo Wu, Haining Li, Cui Cheng, Xiao Han, Xiwen Jiang

**Affiliations:** 1 Guangdong Provincial Key Laboratory of Pharmaceutical Bioactive Substances, School of Basic Medical Sciences, Guangdong Pharmaceutical University, Guangzhou 510006, China; 15913631822@163.com (J.Z.); 13265157396@163.com (W.Q.); 2 College of Biological Science and Engineering, Fuzhou University, Fuzhou 350108, China; baozi61@163.com (B.W.); wzjbshhh@163.com (H.L.);

**Keywords:** diabetic wound, hydrogel, chondroitin sulfate, carbon nanotube, ε-poly-L-lysine, photothermal therapy, antioxidant, conductive dressing

## Abstract

Background: Chronic diabetic wounds, particularly diabetic foot ulcers (DFUs), are prone to recurrent infection and delayed healing, resulting in substantial morbidity, mortality, and economic burden. Multifunctional wound dressings that combine antibacterial, antioxidant, conductive, and self-healing properties may help to address the complex microenvironment of chronic diabetic wounds. Methods: In this study, ε-poly-L-lysine and amino-terminated polyethylene glycol were grafted onto carboxylated single-walled carbon nanotubes (SWCNTs) via amide coupling to obtain ε-PL-CNT-PEG. Aminated chondroitin sulfate (CS-ADH) and a catechol–metal coordination complex of protocatechualdehyde and Fe^3+^ (PA@Fe) were then used to construct a dynamic covalently cross-linked hydrogel network through Schiff-base chemistry. The obtained hydrogels (Gel0–3, Gel4) were characterized for photothermal performance, rheological behavior, microstructure, swelling/degradation, adhesiveness, antioxidant capacity, electrical conductivity, cytocompatibility, hemocompatibility, and antibacterial activity in the presence and absence of near-infrared (NIR, 808 nm) irradiation. Results: ε-PL-CNT-PEG showed good aqueous dispersibility, NIR-induced photothermal conversion, and improved cytocompatibility after surface modification. Incorporation of ε-PL-CNT-PEG into the PA@Fe/CS-ADH network yielded conductive hydrogels with porous microstructures and storage modulus (G′) higher than loss modulus (G′′) over the tested frequency range, indicating stable gel-like behavior. The hydrogels exhibited self-healing under alternating strain and macroscopic rejoining after cutting. Swelling and degradation studies demonstrated pH-dependent degradation, with faster degradation in mildly acidic conditions (pH 5.0), mimicking infected chronic diabetic wounds. The hydrogels adhered to diverse substrates and tolerated joint movements. Gel4 showed notable DPPH• and H_2_O_2_ scavenging (≈65% and ≈60%, respectively, within several hours). The electrical conductivity was 0.19 ± 0.0X mS/cm for Gel0–3 and 0.21 ± 0.0Y mS/cm for Gel4 (mean ± SD, *n* = 3), falling within the range reported for human skin. In vitro, NIH3T3 cells maintained >90% viability in the presence of hydrogel extracts, and hemolysis ratios remained below 5%. Hydrogels containing ε-PL-CNT-PEG displayed enhanced antibacterial effects against *Escherichia coli* and *Staphylococcus aureus*, and NIR irradiation further reduced bacterial survival, with some formulations achieving near-complete inhibition under low-power (0.2–0.3 W/cm^2^) 808 nm irradiation. Conclusions: A dynamic, conductive hydrogel based on PA@Fe, CS-ADH, and ε-PL-CNT-PEG was successfully developed. The hydrogel combines photothermal antibacterial activity, antioxidant capacity, electrical conductivity, self-healing behavior, adhesiveness, cytocompatibility, and hemocompatibility. These properties suggest potential for application as a wound dressing for chronic diabetic wounds, including diabetic foot ulcers, although further in vivo studies are required to validate therapeutic efficacy.

## 1. Introduction

Diabetic foot ulcers (DFUs) are a frequent complication of diabetes and represent a typical chronic wound. According to the International Diabetes Federation, about 10% of the global population had diabetes by 2021, and this number is projected to increase further in the coming decades [1,2,3,4,5]. The prevalence of DFU varies by region, with reported rates of approximately 15.0% in Southeast Asia, 10.0–30.0% in Africa, 21.0% in Brazil, 1.0–17.0% in Europe, and 5.0–20.0% in the Middle East and North Africa [1,2,3,4]. Chronic diabetic wounds such as DFUs are prone to recurrent infection and non-healing, substantially increasing morbidity, mortality, and healthcare costs. Thus, improving diabetic wound management has become a significant challenge for healthcare systems worldwide [3,4,5].

Conventional wound dressings primarily function by absorbing exudate, keeping the wound relatively dry, and serving as a physical barrier to external microorganisms. However, they show limited ability to actively modulate the wound microenvironment, and their efficacy in chronic diabetic wounds is often unsatisfactory. In contrast, hydrogels can absorb exudate, cool the wound surface, relieve pain, and provide a moist environment that favors tissue regeneration while reducing dehydration and scar formation [6,7,8,9]. Hydrogels can also act as carriers for bioactive agents (e.g., antibacterial or antioxidant components), offering opportunities for active wound modulation.

Protocatechualdehyde (PA) is a naturally occurring phenolic aldehyde with reported pro-apoptotic and antibacterial effects [10]. Its aldehyde group can react with amino groups to form imine (Schiff-base) bonds, while catechol groups can coordinate with metal ions such as Fe^3+^ [11]. Through these dynamic covalent and coordination bonds, PA-based systems can give hydrogels self-healing and stimuli-responsive properties, which may improve wound healing [11,12]. Complexes formed by PA and Fe^3+^ (PA@Fe) exhibit near-infrared (NIR) responsiveness and can act as photothermal conversion materials. NIR-triggered photothermal therapy (PTT) has attracted attention for infected wound treatment because it is non-invasive, remotely controllable, and can selectively heat local tissues to kill microorganisms [13,14]. However, excessive laser power may cause thermal damage to surrounding tissues. Therefore, it is important to achieve sufficient antibacterial photothermal effects under relatively low NIR power densities that meet safety guidelines.

Chondroitin sulfate (CS) is a sulfated polysaccharide widely present in the extracellular matrix of human tissues. It can promote cell proliferation, inhibit apoptosis, modulate fibroblast growth factor expression, and reduce inflammatory responses [15]. Owing to its biodegradability, biocompatibility, and safety, CS is often used as a building block for hydrogels. To enhance adhesion and antioxidant properties, CS can be combined with catechol-containing small molecules (e.g., dopamine, DOPA, gallic acid, protocatechualdehyde), enabling dynamic interactions with tissue surfaces.

Electrical properties also play a vital role in wound healing. Skin is sensitive to electrical signals and presents an endogenous electric field (EF) that regulates cell–cell signaling and contributes to wound repair [6,16]. Disruption of the epithelial barrier during wounding can perturb the local EF, thereby impairing cell communication and delaying healing [17]. Conductive wound dressings may help reconstruct local electrical cues without external electrical stimulation. Conductive hydrogels have been shown to influence adhesion, migration, and proliferation of fibroblasts and endothelial cells, partly through activation of PI3K/AKT and MEK/ERK pathways, which are important for angiogenesis [18,19,20].

Carbon nanotubes (CNTs) possess high mechanical strength, large specific surface area, and stable photothermal and electrical properties [21,22,23]. CNT-containing scaffolds have been reported to enhance conductivity, promote cell–cell coupling, and modulate biological activities [24,25,26]. However, pristine CNTs may raise concerns regarding cytotoxicity and hemocompatibility, and surface modification is often required to improve their biological performance.

**Figure 1:**
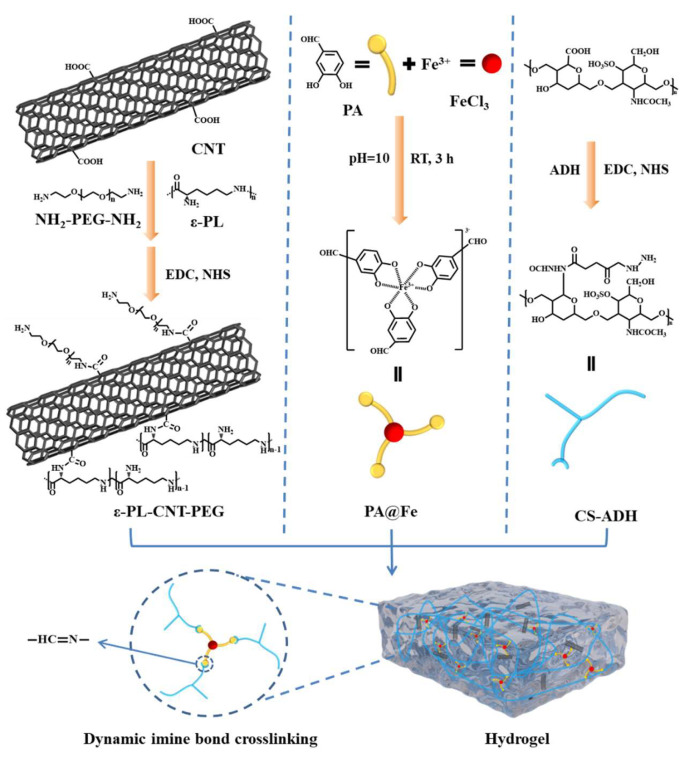
Schematic illustration of the hydrogel system. Synthesis of ε-PL-CNT-PEG by grafting ε-poly-L-lysine (ε-PL) and NH_2_-PEG-NH_2_ onto carboxylated single-walled carbon nanotubes (CNTs), preparation of the PA@Fe complex and aminated chondroitin sulfate (CS-ADH), and formation of a dynamic imine-bonded PA@Fe/CS-ADH/ε-PL-CNT-PEG hydrogel network for diabetic wound dressing.

Based on these considerations, we designed a conductive, photothermal, and antioxidant hydrogel aimed at chronic diabetic wound management. Specifically, we synthesized ε-PL-CNT-PEG by grafting ε-poly-L-lysine (ε-PL) and NH_2_-PEG-NH_2_ onto carboxylated single-walled CNTs. We also prepared aminated chondroitin sulfate (CS-ADH) and PA@Fe complexes. By adjusting the feeding ratios and using Schiff-base crosslinking between aldehydes and hydrazide groups, we constructed a dynamic hydrogel network incorporating ε-PL-CNT-PEG (Figure 1). The resulting hydrogels were evaluated for their physicochemical and biological properties, including photothermal response, antioxidant activity, conductivity, self-healing behavior, cytocompatibility, hemocompatibility, and antibacterial performance under NIR irradiation, to assess their potential for application in chronic diabetic wound care.

## 2. Materials and Methods

### 2.1. Materials

Carboxylated single-walled carbon nanotubes (SWCNTs) and a CNT dispersant were purchased from Nanjing Muke Nanotechnology Co., Ltd. (Nanjing, China). 1-(3-Dimethylaminopropyl)-3-ethylcarbodiimide hydrochloride (EDC), N-hydroxysuccinimide (NHS), chondroitin sulfate (CS, MW = 8000–20,000 Da, sulfate content = 0.2 mmol/g, determined by barium sulfate gravimetry), protocatechualdehyde (PA), Cell Counting Kit-8 (CCK-8), and 2,2-diphenyl-1-(2,4,6-trinitrophenyl)hydrazyl (DPPH) were obtained from Aladdin Reagent Co., Ltd. (Shanghai, China). ε-Poly-L-lysine (ε-PL, MW < 5000 Da) was purchased from Meilun Biotechnology Co., Ltd. (Dalian, China). Adipic dihydrazide (ADH) and NH_2_-PEG-NH_2_ were purchased from Macklin Biochemical Co., Ltd. (Shanghai, China).

All chemicals were used as received unless otherwise stated. Deionized water was used throughout the experiments.

### 2.2. Preparation and Quantification of SWCNT Dispersion

To prepare a CNT dispersion, 51 mg of the CNT dispersant (TNWDIS) was dissolved in 30 mL deionized water by sonication in an ultrasonic cleaning bath for 10 min. Then, 30 mg of SWCNTs was added to the solution and sonicated in an ice bath using a probe-type ultrasonic cell disruptor for a total of 8 h. The resulting suspension was centrifuged at 11,000 rpm for 1 h, and the supernatant containing dispersed SWCNTs was collected.

To determine the SWCNT concentration, 1.5 mL centrifuge tubes were dried overnight in an oven and weighed at room temperature (m_1_). A known volume (V) of the SWCNT dispersion was added to each tube, vacuum dried, and weighed again (m_2_). The concentration C (mg/mL) was calculated as:

$$C=\frac{m_{2}-m_{1}}{V}$$

Each measurement was repeated three times, and the average value was recorded.

### 2.3. Synthesis of ε-PL-CNT-PEG

A portion of the SWCNT dispersion was diluted to obtain 10 mL of solution at 1 mg/mL. Then 10 mg of EDC and 10 mg of NHS were dissolved and slowly added to the SWCNT dispersion under stirring at room temperature for 45 min to activate the carboxyl groups. ε-PL and NH_2_-PEG-NH_2_ were separately dissolved in deionized water at different molar ratios and added dropwise to the activated dispersion (ε-PL first; NH_2_-PEG-NH_2_ after 1 h). The mixture was stirred for 10 h in the dark. The pH was adjusted to 7.0 with 0.1 M NaOH to terminate the reaction.

The reaction mixture was dialyzed against deionized water using a dialysis bag (MWCO 3000 Da) to remove unreacted small molecules and by-products, and then lyophilized to obtain ε-PL-CNT-PEG.

### 2.4. Synthesis of CS-ADH

CS-ADH was prepared via carbodiimide-mediated coupling. Briefly, 1 g of CS was dissolved in 80 mL deionized water under heating and stirring. EDC and NHS were added at a molar ratio of n_(COOH):n_EDC:n_NHS = 1:5:2.5. The pH was adjusted to 5.0 with 0.1 M HCl, and the mixture was stirred in the dark for 1 h to activate the carboxyl groups on CS.

ADH was dissolved in 20 mL deionized water and added to the activated CS solution under stirring. The reaction was continued at room temperature in the dark for 10 h. The pH was then adjusted to 7.0 with 0.1 M NaOH. The reaction mixture was dialyzed (MWCO 3500 Da) against deionized water for 3 days in the dark and then lyophilized for 3 days to obtain CS-ADH as a white cotton-like solid.

### 2.5. Synthesis of PA@Fe Complex

PA was dissolved in deionized water at 80 °C. Freshly prepared FeCl_3_ solution (0.1 M) was added to the PA solution at a molar ratio of Fe^3+^:PA = 1:3. The pH was adjusted to 10.0 using NaOH, and the mixture was stirred at room temperature for 3 h. The resulting PA@Fe complex was lyophilized and stored at room temperature until use.

### 2.6. Preparation of Hydrogels

(1) PA@Fe solution: PA@Fe was dissolved in deionized water to obtain a 0.3 g/mL solution.
(2) ε-PL-CNT-PEG dispersion: ε-PL-CNT-PEG was dispersed in deionized water and sonicated in an ice bath for 1 h with a probe-type ultrasonic cell disruptor. The dispersion was then diluted to 50, 100, 150, and 200 μg/mL.
(3) CS-ADH solution: CS-ADH was dissolved at room temperature in deionized water or in ε-PL-CNT-PEG dispersions of different concentrations to obtain 0.25 g/mL solutions.

PA@Fe and CS-ADH solutions were mixed at predefined volume ratios to form blank hydrogels with different aldehyde-to-amino ratios, denoted as Gel0–3. By adding different amounts of ε-PL-CNT-PEG into Gel0–3 formulations, four hydrogels with varying CNT contents were obtained, denoted as Gel1, Gel2, Gel3, and Gel4. The specific compositions are summarized in Table 1.

### 2.7. Photothermal Performance

For ε-PL-CNT-PEG dispersions, 200 μL of each concentration was placed into 2 mL centrifuge tubes and irradiated with an 808 nm NIR laser (0.8 W/cm^2^). The temperature change over time was recorded using an infrared thermal imaging camera (T4, Dali Technology, Shanghai, China).

For hydrogels, samples were irradiated with 808 nm NIR light at different power densities (0.2 and 0.3 W/cm^2^). Temperature evolution was recorded at predefined time points. Photothermal cycling was evaluated by four consecutive heating–cooling cycles (10 min irradiation followed by 10 min cooling in each cycle).

### 2.8. Rheological Measurements and Self-Healing Tests

Rheological properties were evaluated using a rheometer (MCR302, Anton Paar, Ashland, VA, USA). Frequency sweep tests were performed to measure the storage modulus (G′) and loss modulus (G′′) of Gel0–3 and Gel4 as functions of angular frequency. Temperature sweep tests (heating–holding–cooling) were used to assess the influence of temperature on the mechanical properties.

Self-healing behavior was examined by alternating strain tests. For Gel0–3, oscillatory strain was alternated between 10% and 600%, while for Gel4 it was alternated between 10% and 1500%. Changes in G′ and G″ were monitored over successive cycles to evaluate network disruption and recovery.

Macroscopic self-healing was assessed by cutting cylindrical Gel4 samples into two halves and bringing the cut surfaces into contact at room temperature. The healing process was observed at 5, 10, and 20 min to evaluate recovery of integrity and ability to support their own weight and stretching.

### 2.9. Swelling and Degradation

To evaluate swelling and degradation, lyophilized cylindrical samples of Gel0–3, Gel4, and Gel4 + NIR (Gel4 after NIR irradiation) were immersed in phosphate-buffered saline (PBS) under different conditions: (a) pH 7.4, 37 °C; (b) pH 7.4, 25 °C; and (c) pH 5.0, 37 °C. For each condition, three samples were tested.

At predetermined time points, samples were removed, gently blotted to remove surface water, weighed (W_i_), and returned to the medium. Swelling ratio (%) was calculated as:

$$\mathrm{Swelling}\;\mathrm{ratio}(\%)=\frac{W_{i}-W_{0}}{W_{0}}\times 100\%$$

where W_0_ is the initial dry mass of the hydrogel. Measurements were continued until complete degradation was observed. Each experiment was performed in triplicate.

### 2.10. Adhesion Tests

To qualitatively assess adhesion, 200 μL of hydrogel was applied to various substrates, including paper, metal, plastic, rubber, glass, and porcine skin (or rat skin). After gelation, the samples were gently lifted to observe whether the hydrogel could adhere to and support the substrate without detachment.

### 2.11. Antioxidant Activity

#### 2.11.1. DPPH• Radical Scavenging

Hydrogel samples (200 μL) were incubated with 1 mL DPPH• solution (0.1 mM in ethanol or appropriate solvent) at 37 °C in the dark. At predetermined time points, the supernatant was collected and the absorbance at 517 nm (OD_i_) was measured using a microplate reader. The initial absorbance of DPPH• solution (without hydrogel) was recorded as OD_0_. The DPPH• scavenging percentage was calculated as:

$$\mathrm{DPPH}•\;\mathrm{scavenging}(\%)=\frac{OD_{0}-OD_{i}}{OD_{0}}\times 100\%$$

#### 2.11.2. H_2_O_2_ Scavenging

A calibration curve was constructed by measuring the absorbance (410 nm) of Ti(SO_4_)_2_ (0.03 M, 30 μL) reacted with H_2_O_2_ (100 μL) at known concentrations. For scavenging experiments, 3 mL of H_2_O_2_ solution (1 mM) was mixed with 200 μL hydrogel at 37 °C. At selected time points, 100 μL of supernatant was collected, mixed with 30 μL Ti(SO_4_)_2_, and the absorbance at 410 nm (OD_s_) was measured. The absorbance at 0 min (without scavenging) was recorded as OD_e_. The H_2_O_2_ scavenging percentage was calculated as:

$$H_{2}O_{2}\;\mathrm{scavenging}(\%)=\frac{OD_{e}-OD_{s}}{OD_{e}}\times 100\%$$

### 2.12. Electrical Conductivity

Electrical conductivity of Gel0–3 and Gel4 was measured using a Keithley 6517B electrometer (Keithley Instruments, Solon, OH, USA). Conductivity values were compared with the reported range for human skin (1 × 10^−4^ to 2.6 mS/cm). To visually demonstrate conductivity, hydrogel samples were used as conductive bridges in a simple circuit connecting a power supply and a light-emitting diode (LED); illumination of the LED was used as an indicator of electrical conduction.

### 2.13. Cytocompatibility

Cytocompatibility of ε-PL-CNT-PEG and hydrogel extracts was assessed using NIH3T3 mouse embryonic fibroblasts and the CCK-8 assay. Cells were seeded into 96-well plates at 5 × 10^3^ cells per well and cultured for 24 h to allow attachment. The culture medium was then replaced with medium containing different concentrations of ε-PL-CNT-PEG (50–250 μg/mL) or hydrogel extracts (0.625–10 mg/mL), and cells were incubated for an additional 24 h.

After treatment, medium was removed and replaced with 100 μL of 10% CCK-8 working solution in fresh medium. After 2 h incubation at 37 °C, absorbance at 450 nm was measured. Cell viability (%) was calculated as:

$$\mathrm{Viability}(\%)=\frac{OD_{a}-OD_{0}}{OD_{b}-OD_{0}}\times 100\%$$

where OD_0_ is the absorbance of blank wells (medium without cells), OD_b_ is the control group (cells without ε-PL-CNT-PEG or hydrogel extract), and OD_a_ is the absorbance of treated cells. Experiments were performed in triplicate.

### 2.14. Hemocompatibility

Hemolysis tests were conducted to evaluate hemocompatibility of ε-PL-CNT-PEG and hydrogels. Heparin sodium was dissolved in deionized water (1%) and used to pre-rinse centrifuge tubes. Fresh whole blood from Sprague–Dawley rats was collected and washed three times with 0.9% saline by centrifugation at 3000 rpm for 3 min, discarding the supernatant each time. The red blood cells (RBCs) were resuspended in 0.9% saline to obtain a 2% (*w*/*v*) suspension.

Different concentrations of ε-PL-CNT-PEG (50–250 μg/mL) or hydrogel samples (50 μL) were added to 2 mL centrifuge tubes along with 1 mL RBC suspension and incubated at 37 °C, 60 rpm, for 3 h. After centrifugation (3000 rpm, 3 min), the supernatants were collected, and the absorbance at 540 nm (OD_d_) was measured. Saline (0.9%) served as the negative control (OD_c_), and 0.1% Triton X-100 as the positive control (OD_0_). The hemolysis ratio (%) was calculated as:

$$\mathrm{Hemolysis}(\%)=\frac{OD_{d}-OD_{c}}{OD_{0}-OD_{c}}\times 100\%$$

Each experiment was repeated three times.

### 2.15. In Vitro Antibacterial Assays

Antibacterial activity of ε-PL-CNT-PEG and hydrogels was evaluated against *Escherichia coli* (*E. coli*) and *Staphylococcus aureus* (*S. aureus*). For ε-PL-CNT-PEG, different concentrations were added into wells of a 48-well plate. For hydrogels, 200 μL of each formulation was placed in the wells. Then, 200 μL of bacterial suspension (1 × 10^6^ CFU/mL) was added to each well. Selected wells were irradiated with 808 nm NIR light for 10 min at designated power densities. Subsequently, 500 μL Luria–Bertani (LB) medium was added to each well, followed by incubation at 37 °C for 12 h.

After incubation, 70 μL of bacterial suspension from each well was spread onto LB agar plates and incubated at 37 °C for another 12 h. Colony formation was observed and photographed. Bacterial survival rates were calculated by comparing colony counts of treated groups with those of untreated controls.

## 3. Results and Discussion

### 3.1. Synthesis and Characterization of ε-PL-CNT-PEG and PA@Fe

**Figure 2:**
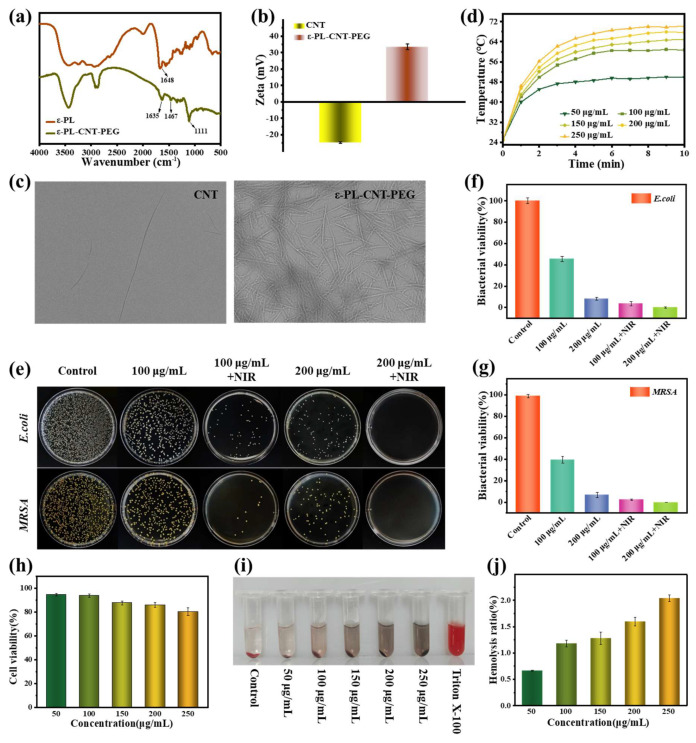
Characterization of ε-PL-CNT-PEG nanoparticles. (**a**) FTIR spectra of CNTs and ε-PL-CNT-PEG. (**b**) Zeta potentials of CNTs and ε-PL-CNT-PEG. (**c**) TEM images showing the morphology and dispersion of CNTs and ε-PL-CNT-PEG. (**d**) Temperature–time curves of ε-PL-CNT-PEG dispersions with different concentrations under 808 nm NIR irradiation (0.8 W/cm^2^). (**e**) Representative infrared thermal images after NIR irradiation. (**f**–**h**) Cell viability of NIH3T3 fibroblasts cultured with different concentrations of CNTs or ε-PL-CNT-PEG for 24 h, determined by the CCK-8 assay. (**i**) Photographs of diluted rat blood treated with saline (negative control), Triton X-100 (positive control), CNTs, and ε-PL-CNT-PEG. (**j**) Quantified hemolysis ratios of CNTs and ε-PL-CNT-PEG at various concentrations. Data are presented as mean ± SD (n = 3).

ε-PL-CNT-PEG was synthesized by grafting ε-PL and NH_2_-PEG-NH_2_ onto carboxylated SWCNTs via EDC/NHS-mediated amidation. Fourier transform infrared spectroscopy (FTIR) showed an absorption band at approximately 1111 cm^−1^, attributed to C–O–C stretching, indicating successful PEG modification (Figure 2a). The zeta potential of SWCNTs shifted from negative to positive after modification, consistent with the presence of ε-PL on the CNT surface (Figure 2b). Transmission electron microscopy (TEM) images showed that ε-PL-CNT-PEG retained a tubular morphology with a high aspect ratio, an average diameter of about 29.1 nm, and lengths of 1–3 μm, and could be uniformly dispersed in water (Figure 2c).

Under 808 nm NIR irradiation (0.8 W/cm^2^), ε-PL-CNT-PEG dispersions exhibited concentration-dependent temperature increases. After 10 min, the maximum temperatures reached approximately 49.8, 60.7, 64.9, 67.7, and 70.2 °C at increasing concentrations, whereas pure water showed negligible temperature change, confirming the photothermal conversion capability of ε-PL-CNT-PEG (Figure 2d).

PA@Fe complexes were confirmed by UV–Vis spectroscopy and Raman spectroscopy. A characteristic absorption peak at 440 nm in the UV–Vis spectrum was attributed to the formation of a stable ternary complex between PA and Fe^3+^ (Figure 2b). Raman spectra showed characteristic peaks in the ranges 470–670 cm^−1^ and 1200–1500 cm^−1^, associated with coordination bonds within PA@Fe. These results indicate successful formation of PA@Fe complexes.

### 3.2. Cytocompatibility and Hemocompatibility of ε-PL-CNT-PEG

The cytocompatibility of ε-PL-CNT-PEG was evaluated using NIH3T3 cells. High concentrations of unmodified CNTs are known to adversely affect cell viability. In this study, at 250 μg/mL, CNTs showed reduced cell viability (~75%) (Figure 2h). In contrast, after modification with ε-PL and NH_2_-PEG-NH_2_, ε-PL-CNT-PEG samples with different concentrations all maintained cell viabilities above 80%. This improvement can be attributed to the hydrophilic PEG chains, which reduce nonspecific interactions with cells and may lower immunogenicity and cytotoxicity.

Hemocompatibility tests showed that the supernatant color in ε-PL-CNT-PEG groups gradually turned darker with increasing concentration, while the positive control (Triton X-100) was bright red and the negative control (saline) was pale yellow (Figure 2i). Quantitative hemolysis ratios for ε-PL-CNT-PEG at various concentrations were 0.7–2.0%, all below the commonly accepted 5% threshold, indicating acceptable hemocompatibility (Figure 2j).

To address the diabetic-wound context, we evaluated inflammatory gene expression in fibroblasts under diabetic-mimicking high-glucose conditions. Cells were cultured in normal glucose (NG, 5.5 mM) or high glucose (HG, 25/30 mM). An osmotic control (NG supplemented with mannitol to match the osmolarity of HG) was included to exclude hyperosmolarity-driven effects. Fibroblasts were treated with extracts of Gel0 or Gel4 for 24 h, and IL-6 and TNF-α mRNA levels were quantified by qPCR using the 2^−ΔΔCt^ method (normalized to NG control).

As expected, HG markedly increased IL-6 and TNF-α expression compared with NG, whereas the osmotic control remained close to NG, indicating that the observed response was primarily glucose-driven (Figure S1). Importantly, hydrogel extracts did not exacerbate the HG-induced inflammatory response. In contrast, Gel0 and Gel4 extracts attenuated HG-induced upregulation of IL-6 and TNF-α, with Gel4 showing a stronger mitigation trend.

### 3.3. Photothermal Performance and Microstructure of Hydrogels

**Figure 3:**
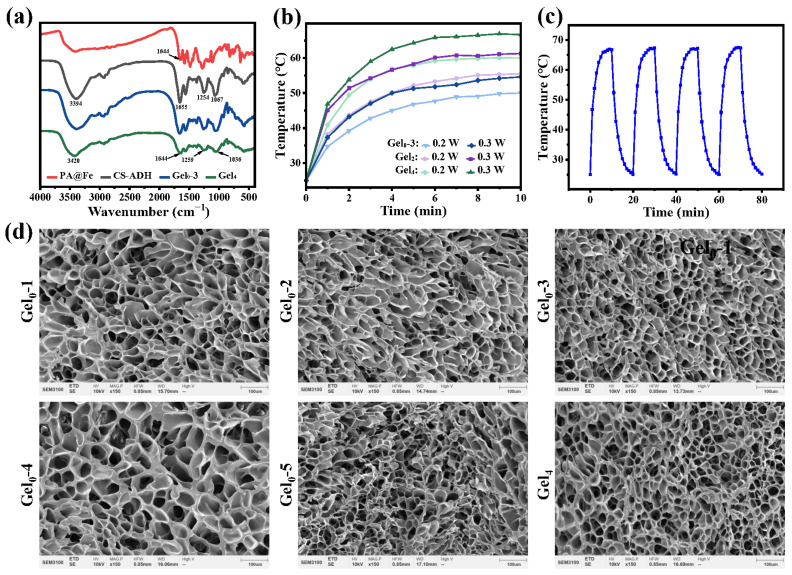
Photothermal performance and microstructure of hydrogels. (**a**) FTIR spectra of CS, CS-ADH, PA@Fe, Gel0–3, and Gel4. (**b**) Temperature–time curves of hydrogels with different ε-PL-CNT-PEG contents under 808 nm NIR irradiation at 0.2 and 0.3 W/cm^2^. (**c**) Photothermal stability of Gel4 over four on/off irradiation cycles (808 nm, 0.3 W/cm^2^). (**d**) SEM images showing the internal porous microstructures of Gel0–3 and Gel4.

Hydrogels containing ε-PL-CNT-PEG (e.g., Gel2, Gel4) exhibited notable temperature rises under 808 nm NIR light at 0.2 and 0.3 W/cm^2^ (Figure 3a). For example, under 0.2 W/cm^2^, maximum temperatures of approximately 50.0, 55.5, and 60.1 °C were observed for gels with increasing CNT content; under 0.3 W/cm^2^, the corresponding temperatures were about 54.6, 61.3, and 66.7 °C (Figure 3b). These results suggest that effective photothermal effects can be achieved under relatively low power densities.

Repeated irradiation–cooling cycles showed that the maximum temperatures remained in a narrow range (approximately 66.7–67.3 °C) over four cycles, indicating stable photothermal conversion and good photothermal stability of the hydrogels (Figure 3c).

SEM images showed that all hydrogels exhibited an interconnected porous architecture (Figure 3d). At the length scale observed (~100 μm), the apparent pore morphology is largely influenced by freezing/drying processes and thus is not used here to quantify molecular crosslinking density. Therefore, SEM is presented as qualitative structural evidence of porosity and continuity of the hydrogel matrix. The mechanical enhancement observed for Gel4 is discussed primarily based on rheology and the CNT-enabled reinforcement/conductive network, while the dynamic coordination and Schiff-base bonds remain the major reversible crosslinking motifs responsible for self-healing.

### 3.4. Rheological and Self-Healing Behavior

**Figure 4:**
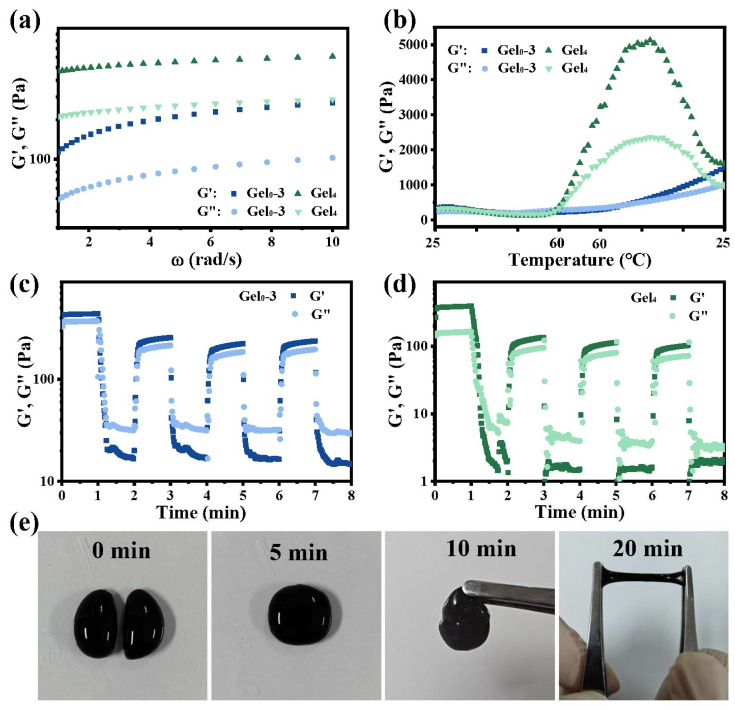
Rheological properties and self-healing behavior of hydrogels. (**a**) Frequency sweep of Gel0–3 and Gel4 showing storage modulus (G′) and loss modulus (G′′). (**b**) Temperature sweep (heating–holding–cooling) of Gel0–3 and Gel4. (**c**) Alternating low (10%) and high (600%) strain tests for Gel0–3 demonstrating disruption and recovery of G′ and G′′. (**d**) Alternating low (10%) and high (1500%) strain tests for Gel4. (**e**) Macroscopic self-healing of Gel4: two pieces brought into contact at room temperature and photographs taken at 0, 5, 10, and 20 min, showing re-adhesion, ability to support its own weight, and stretchability.

Frequency sweep tests showed that G′ was higher than G′′ throughout the tested frequency range for both Gel0–3 and Gel4, confirming gel-like behavior. The storage modulus of Gel4 was higher than that of Gel0–3, suggesting that incorporation of ε-PL-CNT-PEG enhanced the mechanical strength of the hydrogel network (Figure 4a). This enhancement may arise from additional physical and chemical interactions introduced by modified CNTs, including participation of amino and carboxyl groups on CNT surfaces in crosslinking.

Temperature sweep tests (heating–holding–cooling) indicated that both G′ and G′′ increased after thermal treatment, with a more pronounced increase in G′ (Figure 4b). This may be due to enhanced chain mobility and reorganization at elevated temperatures, leading to more homogeneous networks and higher effective crosslinking density. Gel0–3 also showed improved mechanical properties after the thermal cycle, suggesting that the hydrogel structure remained stable within the temperature range used.

Alternating strain tests demonstrated dynamic self-healing behavior. When Gel0–3 was subjected to alternating low (10%) and high (600%) strain, G′ dropped significantly under high strain, indicating network disruption, and recovered upon returning to low strain (Figure 4c,d). Similar behavior was observed for Gel4 under alternating 10% and 1500% strain. Repeated cycles showed that G′ and G′′ could recover to values close to those before high-strain application, indicating that the hydrogels can self-repair after mechanical damage due to the presence of dynamic Schiff-base and coordination bonds.

In macroscopic tests, Gel4 cylinders cut into two pieces could rejoin at room temperature (Figure 4e). After 5 min, no visible cracks were observed at the interface; after 10 min, the healed samples could support their own weight; and after 20 min, they could be stretched without fracture. These results further confirm the self-healing capability of the hydrogels.

### 3.5. Swelling, Degradation, and Adhesion

All hydrogels exhibited a typical swelling–degradation behavior: initial water uptake leading to swelling followed by gradual mass loss and eventual degradation.

**Figure 5:**
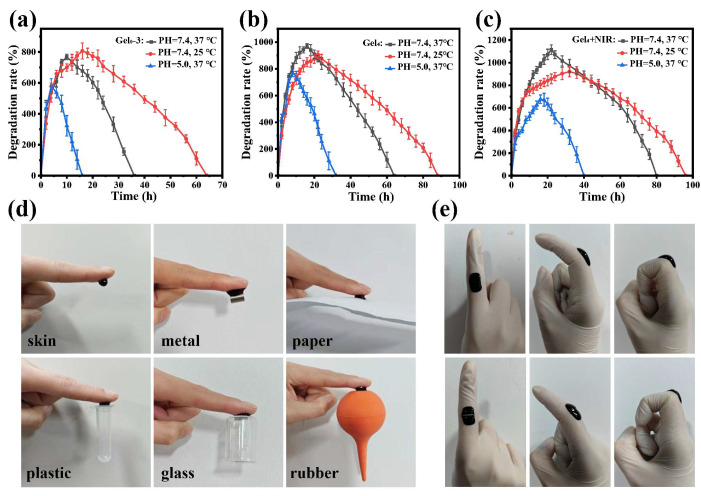
Swelling/degradation behavior and adhesiveness of hydrogels. (**a**) Swelling and degradation profiles of Gel0–3, Gel4, and Gel4 + NIR at pH 7.4 and 25 °C. (**b**) Swelling and degradation at pH 7.4 and 37 °C. (**c**) Swelling and degradation at pH 5.0 and 37 °C. (**d**) Photographs showing adhesion of Gel4 to different substrates, including skin, metal, paper, plastic, glass, and rubber. (**e**) Adhesion and conformability of Gel4 on a finger joint during bending and stretching.

For Gel0–3 at pH 7.4 and 25 °C, maximum swelling was reached at about 16 h, followed by complete degradation at approximately 64 h (Figure 5a). At pH 7.4 and 37 °C, maximum swelling was also reached within 16 h, but degradation was completed by around 36 h, suggesting that higher temperature accelerates network destabilization and degradation. At pH 5.0 and 37 °C, Gel0–3 reached maximum swelling within about 4 h and fully degraded within 16 h, indicating faster degradation in mildly acidic conditions due to the susceptibility of dynamic Schiff-base and coordination bonds to acidic environments.

Gel4 showed a similar trend. At pH 7.4 and 25 °C, maximum swelling occurred at around 22 h, with complete degradation by approximately 88 h (Figure 5b). At pH 7.4 and 37 °C, maximum swelling was reached within 16 h and degradation was completed by 64 h. At pH 5.0 and 37 °C, maximum swelling and complete degradation occurred around 10 h and 32 h, respectively. Compared with Gel0–3, Gel4 displayed faster swelling and higher swelling ratios under the same conditions, likely due to decreased crosslinking density and larger pores after incorporation of photothermal and antibacterial components, which facilitate water uptake and accelerate degradation.

For Gel4 + NIR (Gel4 exposed to 808 nm NIR), at pH 7.4 and 25 °C, maximum swelling was reached at about 36 h, and complete degradation occurred by approximately 96 h (Figure 5c). At pH 7.4 and 37 °C, maximum swelling and degradation occurred at around 22 h and 80 h, respectively. At pH 5.0 and 37 °C, maximum swelling and degradation occurred at around 18 h and 40 h. Compared with Gel4, Gel4 + NIR showed higher swelling and slower degradation, which may be related to increased crosslinking density induced by heat during NIR irradiation. These results indicate that the hydrogels possess pH-responsive and thermally influenced degradation profiles, which could be advantageous for adapting to chronic wound microenvironments.

Adhesion tests showed that the hydrogels could adhere to various substrates, including skin, metal, paper, plastic, glass, and rubber, and support their weight without detachment (Figure 5d). The presence of abundant functional groups (e.g., –OH, –NH_2_, catechol) likely enables multiple interactions (hydrogen bonds, van der Waals forces, and possible coordination) with diverse surfaces. In addition, Gel4 could conform to finger movements and maintain coverage during bending and stretching. Even after partial damage, the self-healing property allowed the hydrogel to recover integrity, potentially extending its practical use time (Figure 5e).

### 3.6. Antioxidant and Conductive Properties

Chronic diabetic wounds continuously generate exudate and reactive oxygen species (ROS), which can exacerbate inflammation and hinder healing. Hydrogels capable of scavenging ROS may help to improve the wound microenvironment.

**Figure 6:**
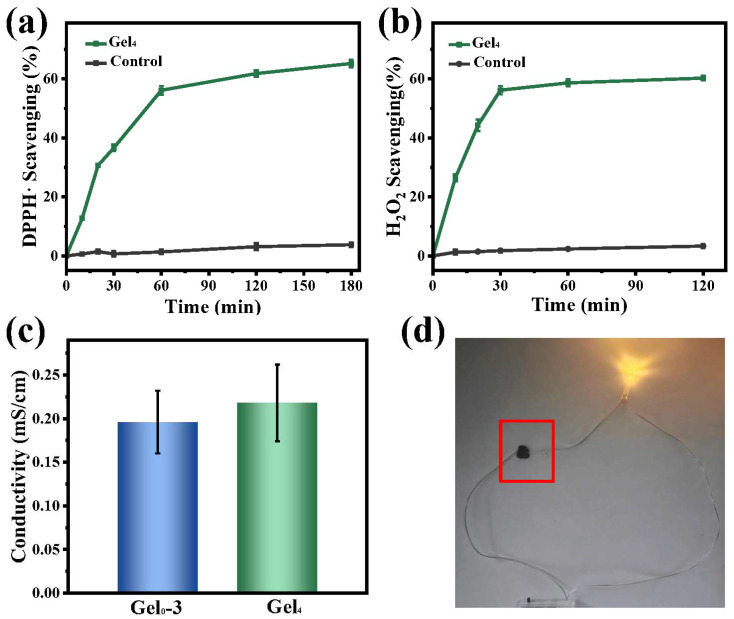
Antioxidant capacity and conductivity of hydrogels. (**a**) DPPH• radical scavenging of Gel4 and control over time. (**b**) H_2_O_2_ scavenging of Gel4 and control over time. (**c**) Electrical conductivity of Gel0–3 and Gel4 measured by a conductivity meter. (**d**) Photograph of an LED circuit in which Gel4 serves as a conductive bridge, demonstrating its electrical conductivity.

DPPH• and H_2_O_2_ scavenging were used to evaluate antioxidant capacity. Gel4 achieved approximately 65% DPPH• scavenging within 3 h and around 60% H_2_O_2_ scavenging within 2 h, suggesting that components such as chondroitin sulfate and catechol groups contribute to ROS scavenging (Figure 6a,b). These antioxidant properties may help attenuate oxidative stress in chronic diabetic wounds.

Electrical conductivity measurements showed that Gel0–3 and Gel4 had conductivities of approximately 0.19 mS/cm and 0.21 mS/cm, respectively, within the range reported for human skin (1 × 10^−4^ to 2.6 mS/cm) (Figure 6c). The increased conductivity in Gel4 is attributed to the incorporation of ε-PL-CNT-PEG. When hydrogels were connected in a simple circuit, the LED successfully lit up, further confirming their ability to conduct electricity (Figure 6d). Conductive dressings with skin-comparable conductivity may facilitate restoration of endogenous electrical signaling and support wound repair.

### 3.7. Cytocompatibility and Hemocompatibility of Hydrogels

**Figure 7:**
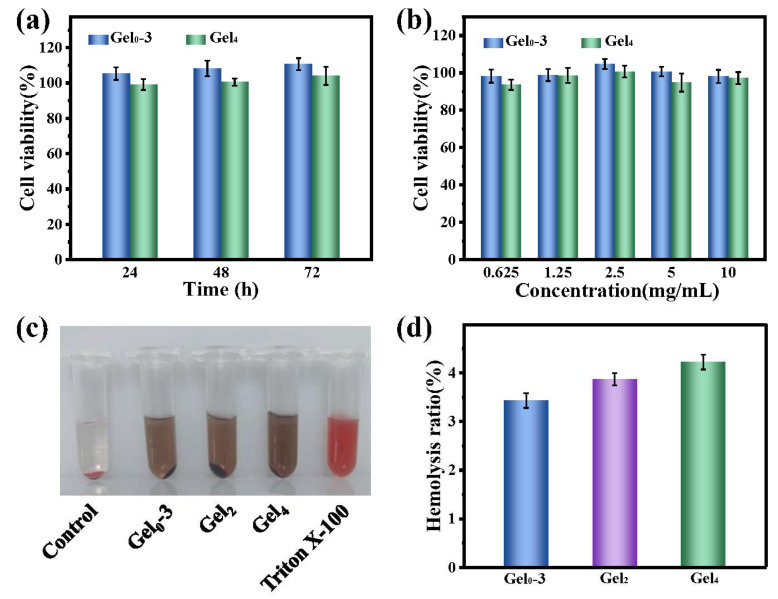
Cytocompatibility and hemocompatibility of hydrogels. (**a**) NIH3T3 cell viability after incubation with Gel0–3 and Gel4 extracts (2.5 mg/mL) for 24, 48, and 72 h (CCK-8 assay). (**b**) Cell viability after 24 h incubation with different concentrations of Gel0–3 and Gel4 extracts. (**c**) Photographs of diluted rat blood treated with saline (negative control), Tukey’s positive control (Triton X-100), Gel0–3, Gel2, and Gel4. (**d**) Hemolysis ratios of Gel0–3, Gel2, and Gel4. Data are presented as mean ± SD (n = 3).

CCK-8 assays showed that NIH3T3 cells cultured with various concentrations of Gel0–3 extracts maintained cell viabilities near 100%, while those treated with Gel4 extracts showed slightly reduced but still high viabilities (>90%) across the tested concentration range (Figure 7a,b). When 2.5 mg/mL hydrogel extracts were applied for different incubation times, Gel0–3-treated cells exhibited viabilities above 100% and Gel4-treated cells above 90% (Figure 7a). The enhanced proliferation may be associated with trace iron ions, which are involved in cellular metabolic and biochemical processes, as well as the antioxidant effects of the PA–Fe network that may support cell growth.

Hemolysis tests indicated that the supernatant colors of hydrogel groups were similar to those of hydrogel extracts (brownish-gray), different from the bright red (positive control) and pale yellow (negative control) (Figure 7c). Quantitatively, hemolysis ratios for Gel0–3, Gel2, and Gel4 were approximately 3.4%, 3.9%, and 4.2%, respectively, all below 5% (Figure 7d). These results suggest that the hydrogels have acceptable hemocompatibility for potential contact with blood.

### 3.8. Antibacterial Activity of Hydrogels

The antibacterial performance of Gel0–3, Gel2, and Gel4 was evaluated against *E. coli* and *S. aureus* under different NIR power densities (0.2 and 0.3 W/cm^2^). After co-culture with bacteria and subsequent plating, the control group showed dense colonies for both strains. Gel0–3 and its NIR-treated groups showed only modest reductions in colony numbers, indicating limited intrinsic antibacterial effect.

**Figure 8:**
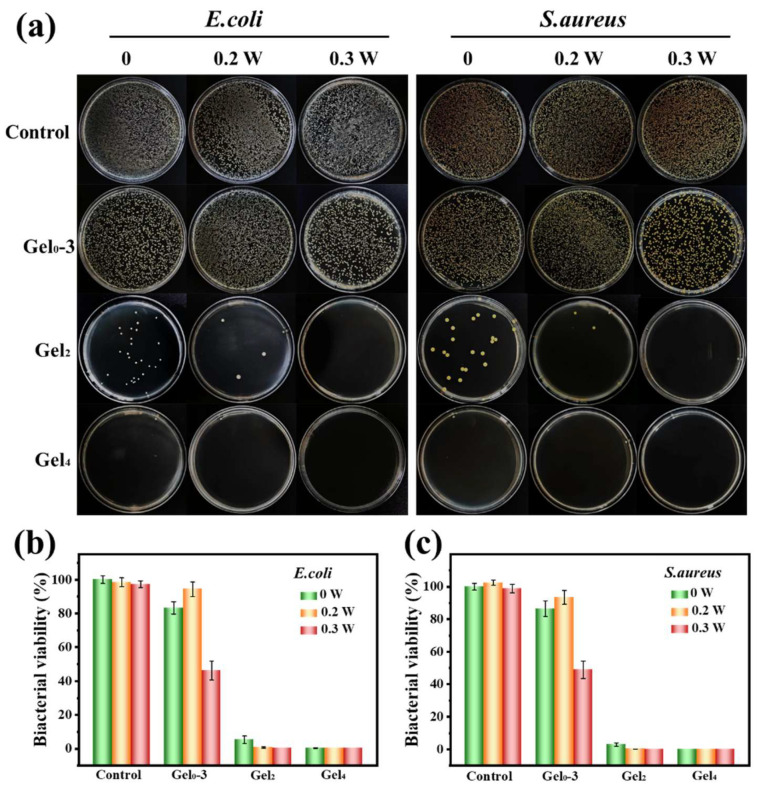
Antibacterial activity of hydrogels with and without NIR irradiation. (**a**) Representative photographs of *E. coli* and *S. aureus* colonies on agar plates after incubation with different hydrogels (Control, Gel0–3, Gel2, Gel4) and 808 nm NIR irradiation at 0, 0.2, or 0.3 W/cm^2^ for 10 min. (**b**) Quantitative residual viability of *E. coli* in each group. (**c**) Quantitative residual viability of *S. aureus* in each group. Data are shown as mean ± SD (n = 3).

In contrast, Gel2 (containing ε-PL-CNT-PEG) showed reduced colony numbers even without NIR, and with 0.3 W/cm^2^ NIR, almost no colonies were observed. All Gel4 groups showed strong antibacterial effects, with no visible colonies under all tested conditions (Figure 8a). Quantitative analysis showed that bacterial survival rates for *E. coli* in the different groups (Gel0–3, Gel0–3 + 0.2 W/cm^2^, Gel0–3 + 0.3 W/cm^2^, Gel2, Gel2 + 0.2 W/cm^2^, Gel2 + 0.3 W/cm^2^, Gel4, Gel4 + 0.2 W/cm^2^, and Gel4 + 0.3 W/cm^2^) were approximately 83.4%, 94.4%, 46.3%, 5.3%, 0.7%, 0.0%, 0.2%, 0.0%, and 0.0%, respectively. For *S. aureus*, the survival rates were about 86.5%, 93.4%, 49.0%, 3.0%, 0.1%, 0.0%, 0.0%, 0.0%, and 0.0%, respectively (Figure 8b,c).

These data indicate that all hydrogels possess some antibacterial activity, but the blank hydrogel (Gel0–3) has limited effect. Incorporation of ε-PL-CNT-PEG markedly enhanced intrinsic antibacterial properties, and the photothermal effect under NIR further improved bactericidal efficacy. Gel4 achieved complete bacterial eradication after 10 min of 808 nm NIR irradiation at 0.2 W/cm^2^, suggesting that effective antibacterial activity can be achieved under a relatively low power density.

## 4. Discussion

In this study, we developed a PA@Fe/CS-ADH/ε-PL-CNT-PEG hydrogel that combines several functions that are considered important for chronic diabetic wound management, namely photothermal antibacterial activity, ROS scavenging, electrical conductivity, adhesion, and self-healing. In vitro, the material showed acceptable cytocompatibility and hemocompatibility, pH-dependent degradation compatible with an infected wound microenvironment, and strong antibacterial activity against *E. coli* and *S. aureus*, especially when combined with low-power near-infrared irradiation. These findings place the present system within the broader class of multifunctional hydrogels that aim to actively modulate the hostile microenvironment of diabetic wounds rather than simply covering the defect [27].

A key design element of this hydrogel is the PA@Fe–CS-ADH dynamic network. Catechol–Fe^3+^ coordination and imine (Schiff-base) bonds have been widely used to endow hydrogels with self-healing, pH/NIR responsiveness, and antioxidant activity. Recent catechol–Fe^3+^ hydrogels have shown that coordination-based networks can efficiently scavenge ROS, display NIR-induced photothermal conversion, and promote skin repair in diabetic wound models [28]. Our results are consistent with these trends: Gel4 exhibited ~60–65% scavenging of DPPH• and H_2_O_2_ within a few hours, and the PA@Fe component contributed to stable photothermal heating under 808 nm irradiation. Importantly, the hydrogel degraded more rapidly under mildly acidic conditions (pH 5.0) than at physiological pH, which is relevant because chronic infected wounds are often slightly acidic. Such pH-sensitive degradation may help the dressing adapt to the wound microenvironment and could facilitate gradual replacement by regenerating tissue.

The incorporation of ε-PL-CNT-PEG distinguishes this system from many previously reported catechol–Fe^3+^ hydrogels. ε-Poly-L-lysine is a cationic peptide already used as a food preservative and investigated as an active antibacterial component in hydrogels and coatings; multiple groups have shown that ε-PL can enhance bacterial killing and, in some cases, promote wound healing in vivo [29]. In the present work, ε-PL is presented in two forms: covalently grafted onto CNTs (ε-PL-CNT-PEG) and as residual free amino groups in the hydrogel network. This design may provide both contact-killing through cationic surface interaction and local release of ε-PL from a dynamic network. At the same time, PEGylation and ε-PL functionalization improved CNT dispersibility and reduced cytotoxicity and hemolysis compared with unmodified CNTs, which addresses one of the main concerns associated with carbon nanotube use in biomedical applications.

The CNT component is also central to the conductive and photothermal behavior of the hydrogel. Conductive dressings are increasingly recognized as promising tools to restore or mimic endogenous electrical cues at wound sites and to interface with electronic monitoring systems [30]. Carbon nanomaterials, including CNTs, are among the most widely used electroactive fillers, and CNT-based hydrogels have been reported to enhance cell migration, support angiogenesis, and accelerate wound closure in experimental models. In our system, small amounts of ε-PL-CNT-PEG raised the conductivity of the hydrogel to ~0.21 mS/cm, within the broad range reported for human skin, while maintaining cell viability above 90%. Although we did not directly demonstrate any bioelectrical regulation of cells, the conductivity and ability to bridge an LED circuit indicate that the material could, in principle, support electrical stimulation or sensing in future studies.

Compared with many photothermal hydrogels that rely on relatively high NIR power densities, an advantage of the present system is that strong antibacterial activity was achieved at 0.2–0.3 W/cm^2^. Some reported photothermal platforms use power densities ≥0.6 W/cm^2^, which raises concerns about thermal damage to surrounding tissues, particularly in poorly perfused diabetic limbs. Here, ε-PL-CNT-PEG and PA@Fe acted synergistically: the intrinsic antibacterial activity of ε-PL and CNTs reduced baseline survival, and photothermal heating further decreased bacterial counts, with Gel4 almost completely eradicating *E. coli* and *S. aureus* after 10 min irradiation at 0.2 W/cm^2^. While these in vitro data cannot be directly extrapolated to in vivo infected wounds, they suggest that combining chemical and photothermal mechanisms may allow effective bacterial control under relatively mild irradiation conditions.

The dynamic nature of the PA@Fe/CS-ADH network also provided self-healing and adhesion, which are increasingly viewed as desirable features for modern dressings. Self-healing hydrogels can conform to moving joints, resist mechanical disruption, and maintain coverage with less frequent replacement, which is particularly relevant for plantar and peri-articular diabetic ulcers. Reviews of self-healing adhesive hydrogels emphasize that catechol-containing systems excel at wet-tissue adhesion and repeated damage–repair cycles [31]. In line with this, Gel4 rejoined within minutes after cutting and adhered to a wide range of substrates, including skin, metal, and plastic, while tolerating finger flexion. These properties may improve patient comfort and reduce the risk of dressing displacement.

Despite these strengths, the present work also has several limitations that should be acknowledged. First, all biological evaluations were conducted in vitro. We did not perform in vivo studies in diabetic or infected wound models, so it remains unknown whether the combined antioxidant, conductive, and photothermal functions translate into faster or higher-quality tissue repair. Many recent multifunctional hydrogels have demonstrated that ROS scavenging and photothermal antibacterial activity can synergistically accelerate re-epithelialization, collagen remodeling, and angiogenesis in diabetic wounds [27]; similar data would be essential to validate the present system. Second, while ε-PL-CNT-PEG greatly improved CNT dispersibility and reduced acute cytotoxicity, long-term biosafety of CNTs, particularly in poorly vascularized chronic diabetic wounds, remains a concern. Systematic studies on CNT retention, potential inflammatory responses, and clearance pathways would be important before considering clinical translation.

Third, our antibacterial assays focused on planktonic *E. coli* and *S. aureus* monocultures. Chronic diabetic wounds, however, often harbor polymicrobial biofilms, including antibiotic-resistant strains and fungi. Several groups have begun to evaluate ε-PL-containing gels or hydrogels in biofilm models and animal infections, showing substantial but sometimes incomplete biofilm reduction [32]. Future work should therefore test the present hydrogel against mixed-species biofilms and in more complex infection models. In addition, we did not investigate how the hydrogel influences key cellular processes such as keratinocyte migration, fibroblast matrix production, macrophage polarization, or endothelial tube formation. These are now standard endpoints in the evaluation of advanced wound dressings and would help clarify the mechanisms by which conductivity, ROS scavenging, and photothermal cues interact at the cellular level.

Finally, compared with emerging “intelligent” dressings that integrate drug delivery, glucose or pH sensing, and wireless monitoring [33,34], our design is still relatively simple. The dynamic imine/coordination network and conductive CNT framework could, in future studies, be combined with glucose-responsive motifs, oxygen-releasing particles, or embedded microelectrodes to provide both therapy and real-time feedback. It may also be possible to tune the mechanical modulus closer to that of native skin and to optimize degradation kinetics for different wound depths and exudate levels.

## 5. Conclusions

In this work, we developed a dynamic conductive hydrogel dressing based on PA@Fe, CS-ADH, and ε-PL-CNT-PEG. The hydrogel integrates reversible dynamic crosslinks with a CNT-enabled conductive/photothermal network, yielding robust mechanical performance, rapid self-healing, strong tissue adhesion, and skin-range electrical conductivity. Moreover, the hydrogel exhibits broad antibacterial activity under NIR irradiation and provides antioxidant capability, which together address key challenges in infected chronic wounds. In vitro cytocompatibility and hemocompatibility tests indicate good biocompatibility.

Although these results support the potential of the hydrogel as an advanced dressing for chronic diabetic wounds (e.g., diabetic foot ulcers), further validation under diabetic-mimicking conditions and in vivo diabetic wound models is needed to confirm therapeutic efficacy and safety. Overall, this multifunctional hydrogel offers a promising platform for next-generation wound dressings.

## Figures and Tables

**Table 1:** Mass percentage of each component in the hydrogel.

Samples	CS-ADH (wt%)	PA@Fe (wt%)	n(-CHO): n(-NH-NH_2_)	ε-PL-CNT-PEG (wt%)	Water Content (wt%)
Gel_0_-1	19.84	0.79	3.5:1	0	79.36
Gel_0_-2	19.80	0.99	4.4:1	0	79.21
Gel_0_-3	19.76	1.19	5.2:1	0	79.05
Gel_0_-4	19.72	1.38	6.1:1	0	78.90
Gel_0_-5	19.68	1.57	7:1	0	78.74
Gel_1_	19.76	1.19	5.2:1	0.02	79.04
Gel_2_	19.76	1.19	5.2:1	0.04	79.02
Gel_3_	19.75	1.19	5.2:1	0.06	79.00
Gel_4_	19.75	1.18	5.2:1	0.08	78.99

## Data Availability

The data and materials supporting the findings are available from the corresponding author upon reasonable request.

## Supplementary Materials

The following supporting information can be downloaded at: https://www.mdpi.com/article/10.3390/polym18030332/s1.
