# Supplementary figures and images for: A Conductive, Photothermal and Antioxidant ε-Poly-L-Lysine/Carbon Nanotube Hydrogel as a Candidate Dressing for Chronic Diabetic Wounds
Source: Polymers (Basel). 2026 Jan 26;18(3):332. doi: 10.3390/polym18030332 (PMC12899480; doi:10.3390/polym18030332)

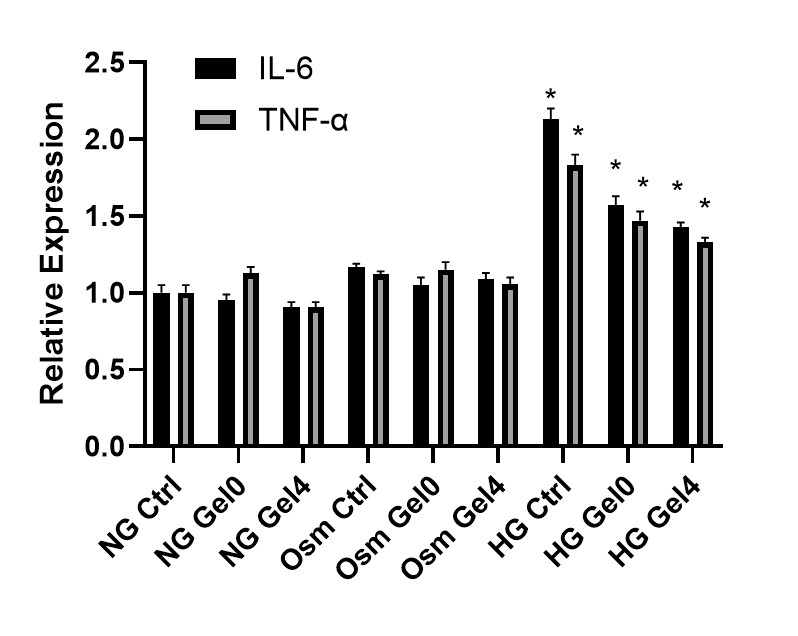

Supplement: Supplementary file 1 [file polymers-18-00332-s001.zip › polymers-4071028-supplementary.jpg]
